# Evaluation of AMG 076, a potent and selective MCHR1 antagonist, in rodent and primate obesity models

**DOI:** 10.1002/prp2.3

**Published:** 2013-09-17

**Authors:** Alykhan S Motani, Jian Luo, Lingming Liang, Jeffrey T Mihalic, Xiaoqi Chen, Liang Tang, Leping Li, Juan Jaen, Jin-Long Chen, Kang Dai

**Affiliations:** Discovery Research, Amgen, Inc.

**Keywords:** Antagonist, diabetes, MCHR1, obesity

## Abstract

Melanin-concentrating hormone (MCH) regulates food intake through activation of the receptor, MCHR1. We have identified AMG 076 as an orally bioavailable potent and selective small molecule antagonist of MCHR1. In mouse models of obesity, AMG 076 caused a reduction in body weight gain in wild-type (MCHR1+/+) but not in knockout (MCHR1−/−) mice. The body weight reduction was associated with decreases in food intake and increases in energy expenditure. Importantly, we show that these MCHR1-dependent effects of AMG 076 were also reflected in improved metabolic phenotypes, increased glucose tolerance and insulin sensitivity. Preliminary data on effects of AMG 076 in obese cynomolgus monkeys are also presented.

## Introduction

Obesity has become a global health epidemic affecting people of virtually all ages and socioeconomic groups. Obesity contributes to the deaths of ∼300,000 adults in the US each year, making it one of the leading causes of preventable mortality (Allison et al. 1999; Baskin et al. 2005; Powell et al. 2011; Wright and Aronne 2011). Importantly, it is now a well accepted risk factor for a number of disorders including type 2 diabetes (Bronner et al. 1995; Chen et al. 2011). However, the few FDA-approved anti-obesity agents such as orlistat (Xenical®, Roche, Basel, Switzerland), have limited efficacy and unpleasant side effects which limit compliance; sibutramine (Meridia®, Abbott Labs, Abbott Park, IL) was withdrawn from markets due to adverse cardiovascular outcomes (Vetter et al. 2010; Bello and Liang 2011). Recently, FDA approved two new weight loss drugs, Qsynia and Bleviq, within a month, emphasizing on the unmet medical need (Holes-Lewis et al. 2013).

Melanin-concentrating hormone (MCH), a neuropeptide highly conserved among vertebrates, plays an important role in the regulation of food intake and energy balance in mammals. MCH is synthesized in the hypothalamus and zona incerta (Nahon 2006) of the brain, areas known to be involved in regulating feeding behaviors (Saito et al. 2000). Acute central administration of MCH stimulates food intake in rodents (Qu et al. 1996) while chronic central infusion leads to obesity and insulin resistance (Della-Zuana et al. 2002; Gomori et al. 2003). MCH expression is increased in fasting and genetically obese animals (Qu et al. 1996; Presse et al. 1996; Tritos et al., 2001; Mizuno et al. 1998; Stricker-Krongrad et al. 2001). Transgenic mice over-expressing MCH in the lateral hypothalamus become obese and are susceptible to insulin resistance on a high-fat diet (Ludwig et al. 2001). In contrast, animals lacking expression of MCH through targeted deletion of the gene are lean, hypophagic, and hypermetabolic (Shimada et al. 1998). These data suggest that MCH activates neuronal pathways, resulting in increased food intake and altered energy metabolism.

Only one MCH receptor, MCHR1, is expressed in rodents. MCHR1 was identified as a G protein-coupled receptor (GPCR; Kolakowski et al. 1996; Saito et al. 2000; Hervieu 2003). In support of the role of MCH/MCHR1 signaling in the regulation of food intake and energy balance, MCHR1 knockout mice (MCHR1−/−) are lean, have an increased metabolic rate and are resistant to diet-induced obesity (Marsh et al. 2002; Kokkotou et al. 2005). Unlike rodents, humans and some species of nonhuman primates (NHP) express a second MCH receptor, MCHR2 (An et al. 2001; Hill et al. 2001; Sailer et al. 2001; Tan et al. 2002). The biological role of MCHR2 is not clear and the relative contributions of MCHR1 and MCHR2 to MCH-mediated effects are little understood.

In this article, we describe results of evaluation of AMG 076, a potent and selective small molecule antagonist of MCHR1 (Mihalic et al. 2012), in rodent and NHP models of obesity.

## Materials and Methods

### Compounds

The MCHR1 selective antagonist AMG 076, 1-[2-((4aR, 11R, 11aS)-11-Methyl-9-trifluoromethyl-1,3,4,4a,5,6,11,11a-octahydro-pyrido[4,3-b]carbazol-2-yl)-ethyl]-cyclohexane carboxylic acid was synthesized at Amgen (Mihalic et al. 2012). Sibutramine was prepared from disassembled capsules of Meridia®.

### Radioligand binding and Ca^2+^ mobilization assays

Radioligand membrane binding and aequorin assays were carried out as described previously (An et al. 2001). [^125^I]-MCH (2200 Ci//mmol) was purchased from PerkinElmer (Waltham, MA) and MCH peptide from Phoenix Pharmaceuticals (Burlinggame, CA). Inhibition of MCH-induced Ca^2+^ mobilization was assayed in HEK293 cells stably expressing human MCHR1 (HEK293-MCHR1), using a FLIPR assay kit from Molecular Devices (Sunnyvale, CA) according to the manufacturer's protocol. IC_50_ was determined by nonlinear regression curve fitting program (GraphPad Prism). Inhibition of MCH-induced Ca^2+^ mobilization in multiple species was measured by aequorin assay in CHO cells transiently expressing MCHR1 of different species. Concentrations of MCH used for the FLIPR and aequorin assays were 12 nM and 35 nM, respectively. All assays were repeated at least three times for K_i_ and IC_50_ determination and representative experiments are presented.

The selectivity of AMG 076 was evaluated in radioligand displacement assays against a panel of 64 GPCRs, transporters and ion channels by Eurofins Panlabs (Taipei, Taiwan). The affinity of AMG 076 for 5HT2C was also evaluated by Amgen in radioligand displacement-binding assay using 5HT2C membrane and specific radioligand [^3^H]-Mesulergine purchased from PerkinElmer (Waltham, MA).

### Mouse models

Mice were cared for in accordance to the Guide for the Care and Use of Laboratory Animals (Institute for Laboratory Animal Resources 1996). Animals were (group or singly housed, as described below) at an AAALAC, Intl-accredited facility in autoclaved positive pressure ventilated microisolator housing. All research protocols and animal housing was approved by Amgen San Francisco Institutional Animal Care and Use Committee (IACUC).

Animals had ad libitum access to feed (as described below) and water (reverse osmosis-purified) via automatic watering system and water bottle. Animals were maintained on a 12:12 h light: dark cycle in rooms at (state temp range, and humidity range), except as described for energy expenditure studies (described below). All animals were determined specific pathogen-free ones (full list is described at http://jaxmice.jax.org/health/agents_list.html).

Mice with targeted disruption of MCHR1 backcrossed fully to C57BL/6 (MCHR1−/−; Roy et al. 2006) were bred in-house (heterozygous matings), genotyped, and then individually identified by electronic ID chips (AVID, Norco, CA) placed in the intrascapular region at the time of weaning.

For the obesity prevention study, 5–6 week old singly housed male and female MCHR1 knockout (−/−), heterozygous (+/−) or wild-type mice (+/+) were randomized into treatment groups by date of birth, gender, and genotype. AMG 076 (at concentrations in the diet determined to deliver 3, 10, and 100 mg kg^−1^ day^−1^) was prepared as admixture in diet (60 kcal% fat;. Research Diets D12492i, New Brunswick, NJ) and mice were transferred to this high-fat diet on initiation of the study. Food intake and body weight were monitored for the duration of the study. Fat mass was measured by dual energy x-ray absorptiometry (DEXA) imaging.

High-fat diet-induced obese (DIO) mice were generated as follows: male C57BL/6 (JAX-WEST, Sacramento, CA) mice weaned at 4–5 weeks of age were maintained on a high-fat diet (60 kcal% fat, Research Diets D12492i; New Brunswick, NJ) for 20–24 weeks. Prior to the start of dosing, group-housed mice were randomized into treatment groups based on body weight. Induction of hyperglycemia and insulin resistance was confirmed by comparing baseline glucose and insulin levels with chow fed (Harlan Teklad 2918) lean, control mice. AMG 076 or sibutramine were suspended in vehicle (1% Tween-80 [v/v], 1% carboxymethyl cellulose [w/v], in milliQ water) and administered by oral gavage (5–10 mL/kg) at the doses indicated.

### Glucose tolerance and insulin sensitivity

Glucose tolerance was determined in overnight (6 pm to 9 am) fasted mice challenged with 0.75 g/kg of an oral bolus of D-glucose (7.5% in water). Insulin sensitivity was determined in 4-h (from 6 am to 10 am) fasted mice, challenged with 1.5 U/kg of an intraperitoneal bolus of human insulin (0.15 U/mL). Blood was sampled from conscious mice by tail nick for measurement of insulin (ALPCO diagnostics, Windham, NH), leptin (R&D Systems, Minneapolis, MN), triglycerides, total cholesterol, and NEFA (Hitachi Clinical Analyzer, Tokyo, Japan). Whole blood glucose was measured using a glucose meter (Accu-Check Blood Glucose Monitoring System, Roche, Basel, Switzerland).

### Energy expenditure

Female mice (MCHR1−/−, MCHR1+/− or MCHR1+/+) were individually housed in a 4-chamber, indirect open circuit calorimeter (Oxymax, Columbus Instruments, OH) at 28°C (approximating thermoneutrality) and provided with free access to food and water. Mice were acclimated in the chambers for 24 h prior to initiation of data collection over the subsequent 48-h period. AMG 076 or sibutramine were administered by oral gavage (5–10 mL/kg) at the doses indicated, once daily approximately 90–120 min prior to initiation of the dark cycle (from 6 pm to 6 am).

### Primate model

The chronic effects of AMG 076 were evaluated in spontaneously obese cynomolgus monkeys. Cynomolgus monkeys (Macaca fascicularis, Kunming, China) were cared for in accordance with the Guide for the Care and Use of Laboratory Animals (Institute for Laboratory Animal Resources 1996). Animals were housed at an indoor facility in species-specific housing on a 12:12 h light: dark cycle in rooms maintained at temperature of 18–26°C with a relative humidity of 60–80% following AAALAC guidelines. All research protocols and animal housing was approved by the Yunnan Laboratory Primate Institute IACUC.

Male spontaneously obese monkeys of 8–15 years old, weighing 7–13 kg, were screened for health and body weight for enrollment into the study. A fixed amount of certified primate diet (Kunming, China) established during the acclimation period, was given to the animals daily throughout the course of the study. The feeding times were from 0900 h to 1100 h (±30 min) and then between 1500 h and 1700 h (±30 min). In addition, apple slices (150 g/animal) were provided to the animals between 1200 h and 1300 h (±30 min). Animals had ad libitum access to city water, via an automatic watering system.

After acclimation to doing and handling for at least 3 weeks, monkeys were randomized into four treatment groups based on baseline food consumption, body weight, body mass index (BMI), and abdominal fat content (determined by computed tomography [CT], described below). Animals received a twice daily oral bolus of either vehicle (1% Tween-80 [v/v], 1% carboxymethyl cellulose [w/v], in milliQ water) or AMG 076 (0.3, 1.0, or 3.0 mg/kg, bid) for 90 days. The daily food consumption and behavioral observations were conducted throughout the duration of study. Weekly body weight was measured. BMI was calculated by body weight (kg) divided by the square of crown-rump length. Intra-abdominal fat content was determined using CT scan (Siemens, Somatom Esprit+) on day 3 and day 88. For each monkey, 10 scans at 0.8 cm intervals, 5 cm above and 3 cm below the level of the umbilicus, were obtained. Monitoring was continued for 4 weeks after AMG 076 administration was discontinued. No gross or clinical abnormalities were observed in response to treatment or handling during the course of the study.

### Statistics

Statistical analysis was performed by analysis of variance (ANOVA) followed by a two-sample t-test with Bonferroni correction. Long-term body weight changes in the DIO mice with different treatments (Fig. 4A) were analyzed using Dunnett's test to compare treatments to vehicle control using SAS process general linear model (SAS proc glm, SAS Institute, Cary, NC). Data are shown as mean ± SEM (standard error of mean).

## Results

### AMG 076 is a potent and selective small molecule antagonist of MCHR1

**Figure 1:**
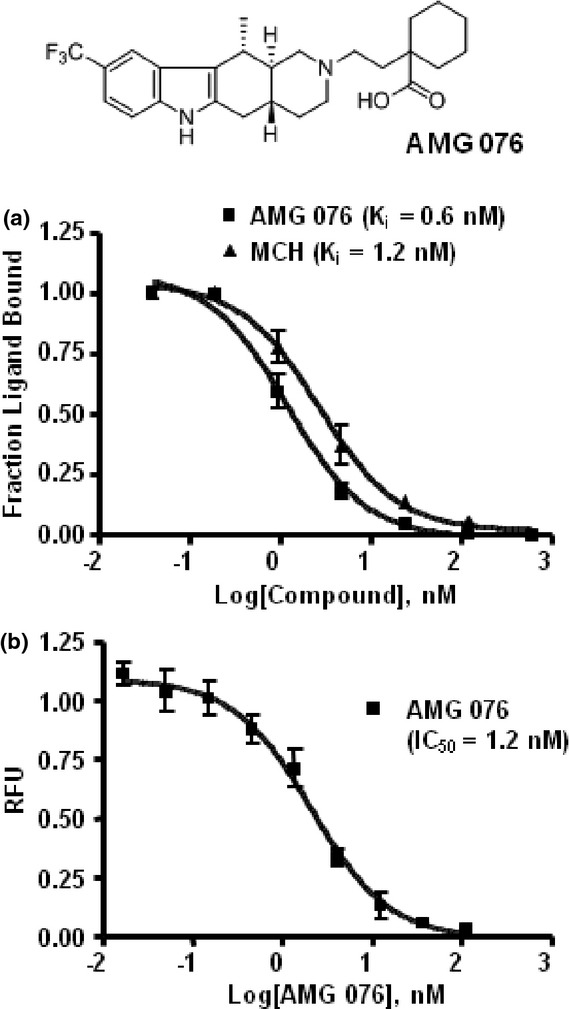
Binding affinity and functional antagonism of AMG 076 to MCHR1. (A) Binding affinity of AMG 076 to MCHR1 was determined by [^125^I]-MCH displacement membrane-binding assay. The human MCHR1 membrane was prepared from HEK293 cells stably expressing human MCHR1 (HEK293-MCHR1). Unlabeled MCH was included as a reference. (B) Functional antagonism was determined by FLIPR Ca^2+^ mobilization assay. MCH at EC_50_ (12 nmol/L) was used to stimulate Ca^2+^ response in the presence of AMG 076 at the concentrations as indicated. RFU, relative fluorescence unit.

AMG 076 was discovered through high throughput screening and lead optimization at Amgen (Mihalic et al. 2012). The compound displaces [^125^I]-MCH with a K_i_ of 0.6 ± 0.10 nM (Fig. 1A). Functional antagonism of AMG 076 was demonstrated through inhibition of the MCH-induced Ca^2+^ mobilization in HEK293-MCHR1 cells with an IC_50_ of 1.2 ± 0.26 nmol/L (Fig. 1B). AMG 076 showed similar activity for MCHR1 across animal species as determined by aequorin assay in cells transiently expressing MCHR1 of different species (Mihalic et al. 2012).

AMG 076 is highly selective against MCHR2 and showed no significant inhibitory activity at >10,000 nmol/L in FLIPR Ca^2+^ mobilization assay (Fig. 1B). The selectivity of AMG 076 was further evaluated in a screen by Eurofins PanLabs against a panel of GPCRs, transporters and ion channels using radioligand displacement assays. IC_50_ for all of the 64 targets screened except for 5HT2C was >2000 nmol/L. K_i_ of AMG 076 for 5HT2C was determined to be 1120 ± 59 nmol/L, >1000-fold higher than that for MCHR1.

### AMG 076 reduced body weight gain in mice on high-fat diet

**Figure 2:**
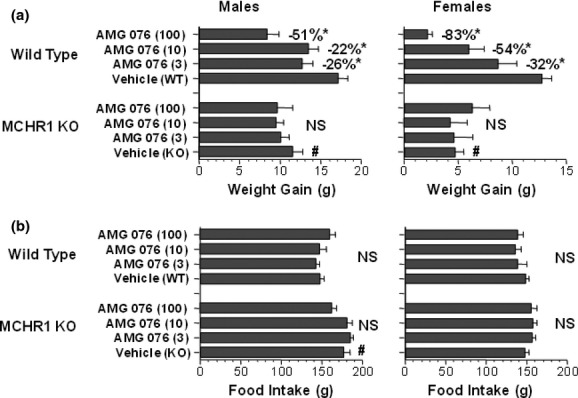
AMG 076 reduced body weight gain in male and female mice fed a high-fat diet. (A) Body weight. (B) Food intake. AMG 076 (3, 10, and 100 mg kg^−1^ day^−1^) was prepared as an admixture high-fat diet and administered ad libitum to C57BL/6 mice with wide-type (WT) MCHR1(+/+) or inactivated (KO) allele of MCHR1 (MCHR1(−/−)). Doses are indicated in mg kg^−1^ day^−1^. Responses are indicated as mean ± SEM of *n* = 7–9 mice per group. *P < 0.05 versus Vehicle (WT). #*P* < 0.05 versus Vehicle (WT); NS, not significant versus Vehicle (KO).

**Figure 3:**
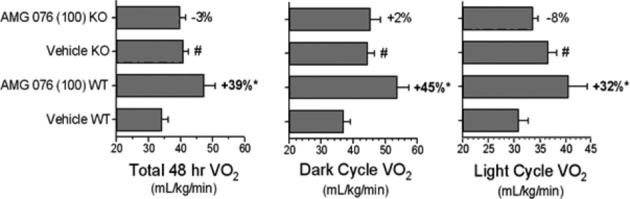
AMG 076 increased oxygen consumption in mice. Energy expenditure in a sample population of AMG 076-treated C57BL/6 female mice from the study in Fig. 2 was measured as described in Materials and Methods. AMG 076 and sibutramine were suspended in vehicle and administered by oral gavage (5–10 mL/kg) at the doses indicated, once daily approximately 90–120 min prior to initiation of the dark cycle (6 pm to 6 am). Doses are indicated in mg kg^−1^ day^−1^. Responses (after 20 weeks of dosing) are indicated as mean ± SEM of *n* = 8 mice per group. *P < 0.05 versus vehicle WT. #*P* < 0.05 versus vehicle WT. Values indicate percent change from respective vehicle genotype controls.

AMG 076 was evaluated for its ability to prevent the accumulation of body fat in C57BL/6 mice transferred onto a high-fat diet. To confirm that the effects of AMG 076 were mediated specifically through MCHR1, MCHR1(−/−) mice with the same genetic background (Roy et al. 2006) were evaluated in parallel. After 8 weeks of treatment with AMG 076, a dose-related reduction in weight gain was observed specifically in wild-type mice but not in MCHR1(−/−) mice (Fig. 2). Reduced body weight gain in MCHR1 wild-type (+/+) mice was associated with a reduction in accumulated fat mass, without loss of lean mass (data not shown). Under these conditions, however, treatment with AMG 076 did not significantly affect food intake in either MCHR1(+/+) or MCHR1(−/−) mice. A reduction in weight gain in the absence of a change in food intake in AMG 076-treated MCHR1(+/+) mice suggested a possible increase in energy expenditure, which is a notable phenotype of the MCHR1(−/−) mice (Marsh et al. 2002). Oxygen consumption (VO2) in MCHR1(−/−) versus MCHR1 (+/+) mice was compared after 20 weeks of compound treatment (between days 143 and 162 of study) using indirect calorimetry. MCHR1(−/−) mice dosed with vehicle exhibited a higher VO2 compared with MCHR1(+/+) mice receiving vehicle (Fig. 3). Furthermore, treatment of the MCHR1(+/+) mice with AMG 076 (100 mg kg^−1^ day^−1^) was associated with a significant increase in measured VO2 compared with the MCHR1(+/+) mice that received vehicle. In contrast, no effect of AMG 076 (100 mg kg^−1^ day^−1^) treatment was observed in the MCHR1(−/−) mice compared with the MCHR1(−/−) mice that received vehicle. In summary, these data show that the prevention of high-fat diet-induced body weight gain by AMG 076 is MCHR1-dependent and is at least partly mediated through an increase in energy expenditure.

### AMG 076 reduced body weight gain and food intake in DIO mice

**Figure 4:**
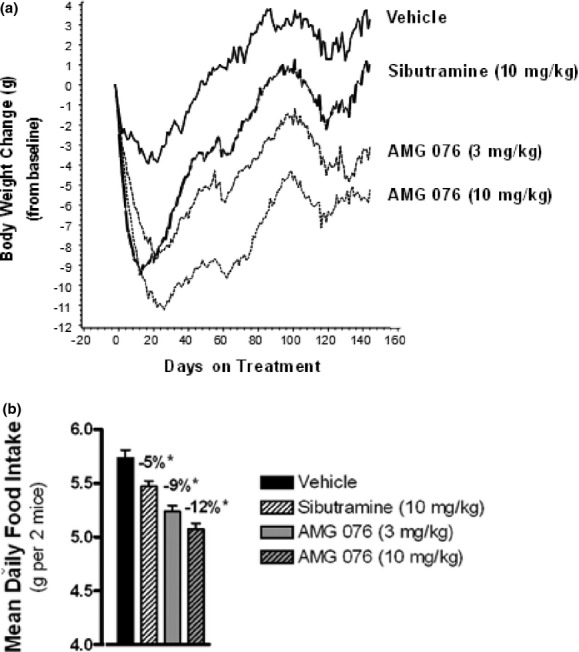
AMG 076 inhibited weight gain and food intake in DIO mice. Pair-housed, high-fat diet-induced obese (DIO) male C57BL/6 mice were maintained on a high-fat diet for 20–24 weeks prior to AMG 076 dosing. Test compounds were administered once daily by bolus oral gavage (5–10 mL/kg) at the indicated doses and body weight (A) and food intake (B) were monitored daily. (A) Sibutramine, *P* < 0.05 versus vehicle between day 2–34 and day 47–80. AMG 076 (3 mg/kg), *P* < 0.05 versus vehicle between day 7–144. AMG 076 (10 mg/kg), *P* < 0.05 between day 3–144. (B) *P < 0.05 versus vehicle. Values indicate percent change from Vehicle. Responses are indicated as mean ± SEM, *n* = 10 mice per group.

The diet-induced obese (DIO) mouse model is a well-established animal model for testing anti-obesity agents (Rossmeisl et al. 2003). In particular, these mice exhibit metabolic changes (hyperglycemia, hyperinsulinemia, glucose intolerance, insulin resistance) and share some clinical characteristics of obese patients with metabolic syndrome. Male C57BL/6 mice were maintained on a high-fat diet for at least 20 weeks prior to the start of drug administration, which continued for an additional 20 weeks. The treatments consisted of vehicle, AMG 076 (3 and 10 mg kg^−1^ day^−1^) or sibutramine (10 mg kg^−1^ day^−1^). The effect of AMG 076 and sibutramine on body weight was assessed over the period from day 0 to 144 (Fig. 4A). Reductions in body weight for sibutramine and AMG 076 (both 3 and 10 mg kg^−1^ day^−1^) reached statistical significance by day 2, 7, and 3, respectively, and remained statistically significant through day 144. Dose-related exposures of AMG 076 in plasma were confirmed as summarized in Table S1.

Food intake measurements were initiated 3 weeks into the study and continued through day 115 of treatments. A dose-related reduction was observed in daily food intake in response to AMG 076 treatment relative to vehicle during the course of treatment (Fig. 4B; *P* < 0.05 for both doses). Sibutramine also reduced food intake (*P* < 0.05), but to a lesser extent than AMG 076.

### AMG 076 showed anti-diabetic effects in DIO mice

**Figure 5:**
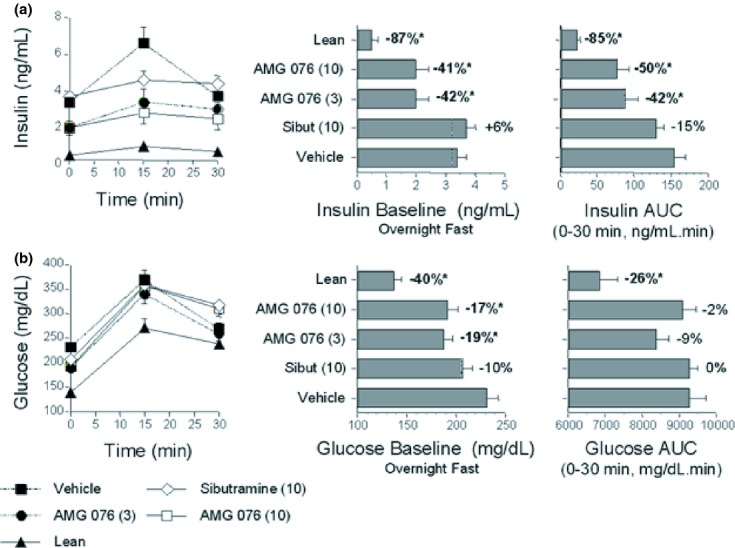
AMG 076 reduced fasted glucose and insulin levels and insulin levels in response to a glucose challenge. (A) Insulin levels. (B) Glucose levels. On day 130 of compound treatment the DIO male C57BL/6 mice were challenged with an oral glucose bolus (0.75 g/10 mL/kg) after an overnight fast. Blood was sampled from the tails of conscious mice. Responses are indicated as mean ± SEM, n = 6–8 mice per group. Values indicate percentage change from vehicle. Test material doses are indicated in mg kg^−1^ day^−1^. *P < 0.05 versus vehicle. AUC, area under the concentration curve over time course as indicated; Sibut, sibutramine.

**Figure 6:**
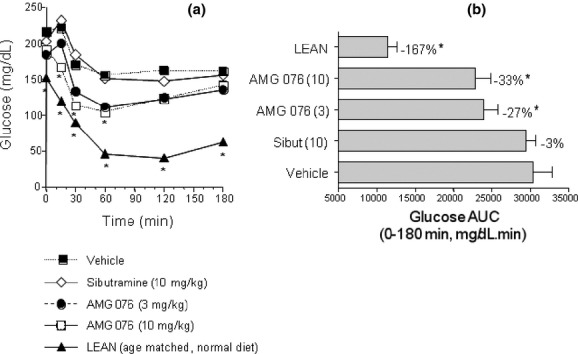
AMG 076 increased the glucose-lowering response to a bolus of insulin. On day 95 of compound treatment the DIO male C57BL/6 mice were fasted for 4 h and then challenged with insulin (1.5 U/10 mL/kg) intraperitoneally. Blood was sampled at time points post insulin injection as indicated. (A) Whole blood glucose concentrations. (B) Areas under the whole blood glucose concentration curves (Glucose AUC). Responses are indicated as mean ± SEM, *n* = 6–8 mice per group. Test material doses are indicated in mg kg^−1^ day^−1^. Sibut, sibutramine. *P < 0.05 versus vehicle.

Anti-diabetic effects of AMG 076 were evaluated in DIO mice. Chronic administration of AMG 076 resulted in a significant decrease in fasting insulin and glucose levels, and increases in glucose tolerance (Fig. 5) and insulin sensitivity (Fig. 6). In contrast, no improvement in these aforementioned parameters was observed in sibutramine-treated DIO mice. Thus, AMG 076-mediated changes in body weight and food intake was reflected in the improvement of the metabolic phenotype of these mice.

### Effect of AMG 076 on obesity-related parameters in obese cynomolgus monkeys

Similar to humans, cynomolgus monkeys express both MCHR1 and MCHR2 and represent a debatably more relevant animal model for evaluating antagonists of the MCH pathways. In this study, AMG 076 appeared well tolerated in the spontaneously obese cynomolgus monkeys. No abnormal clinical observations or behavioral changes were noted and treatments did not affect clinical chemistry and hematology measurements (data not shown).

**Figure 7:**
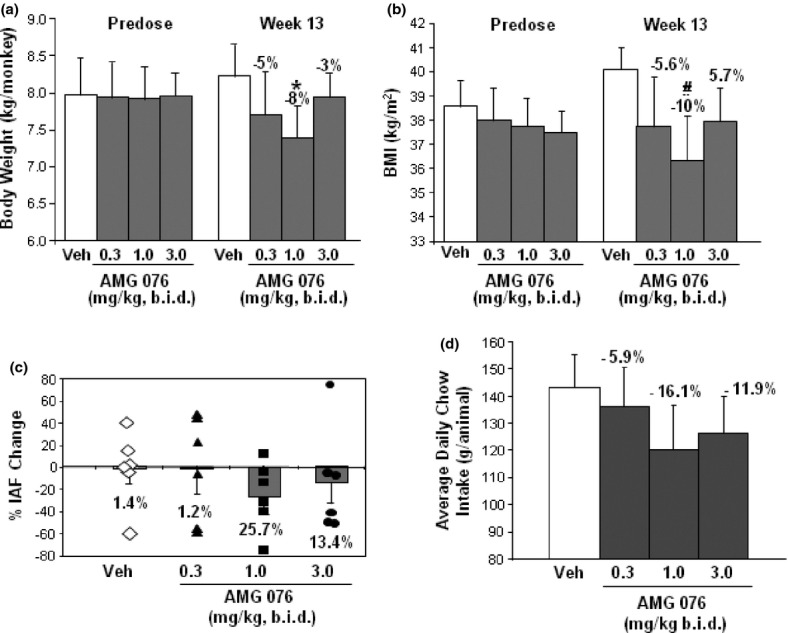
Effect of AMG 076 on body weight in obese cynomolgus monkeys. Spontaneously obese male cynomolgus monkeys received twice daily oral gavage of either vehicle or AMG 076 for 90 days. Body weight and BMI were measured weekly, food intake daily, intra-abdominal fat before and after the treatment. (A) Body weight and BMI are indicated as the mean ± SEM. *P < 0.05 versus vehicle; #*P* < 0.05 versus vehicle. (B) Intra-abdominal fat. ♢Vehicle treatment group; AMG 076 treatment groups: ■0.3 mg/kg, ▲1 mg/kg, ●3 mg/kg. (C) Daily chow intake. The percent change relative to pretreatment values calculated from individual measurements, *n* = 6 monkeys per group.

In response to treatment with AMG 076, body weight, and BMI trended down in the AMG 076 treatment groups compared with those of the vehicle group (Fig. 7A and B). Statistic significance, however (*P* < 0.05), was reached only by the 1 mg/kg group. Five of the six monkeys given the vehicle gained significant weight, and at least three monkeys in each of the AMG 076 dose groups showed a commensurate decrease in body weight and BMI. Intra-abdominal fat (IAF) content was measured on day 88 of the treatment using abdominal CT scan and food intake was measured daily. Although a downtrend in IAF and food intake similar to that of body weight and BMI was observed with the AMG 076 treatment groups, the changes did not reach statistical significance by any of the treatment groups (Fig. 7C and D).

## Discussion

AMG 076 has been identified as a potent and selective MCHR1 antagonist. Chronic administration of AMG 076 resulted in significant reduction in body weight gain in nonobese mice fed a high-fat diet and in DIO mice. A reduction in food intake was also detected in the already obese DIO mice but not in those mice that were not obese at the start of treatment. Energy expenditure was, however, significantly increased in both the nonobese and the DIO mice. Therefore, inhibition of food intake and increased energy expenditure may both contribute to the reduction in body weight from AMG 076 treatment. These effects of AMG 076 on body weight and food intake are believed to be mediated through MCHR1 as they were absent in the MCHR1(−/−) mice treated with AMG 076 up to 100 mg kg^−1^ day^−1^. Although the binding of AMG 076 to MCHR1 in the brain of the treated animals was not measured, the dependence of the effects of AMG 076 on MHCR1 is in support of AMG 076 reaching and antagonizing MCHR1 effectively. In addition, these results are consistent with those of previously reported animal studies of the MCH/MCHR1 system and MCHR1 antagonists (Chen et al. 2002; Marsh et al. 2002; Zheng et al. 2005; Ito et al. 2010; Chung et al. 2011).

We did not observe significant effect of AMG 076 treatment on food intake in lean mice on high-fat diet. We, however, detected a significant reduction in food intake in DIO mice treated with AMG 076. These observations suggest that different metabolic state of the animals may be critical in determining how antagonism of MCHR1 may contribute to body weight loss, through increasing metabolic rate or suppressing appetite, or both.

Obesity has been shown to be a risk factor for type 2 diabetes and is known to exacerbate diabetes in humans (Chen et al. 2011). We observed that chronic AMG 076 treatment of obese mice led to significantly lowered baseline glucose and insulin levels and an increase in glucose tolerance and insulin sensitivity compared with vehicle- and sibutramine-treated mice. These findings are consistent with observations in MCHR1 or MCH deficient mice (Chen et al. 2002; Bjursell et al. 2006; Jeon et al. 2006) and emphasize potential antidiabetic benefits of antagonism of MCHR1.

Rodents do not express MCHR2 (Tan et al. 2002). Interestingly, MCHR2 maps to a region on chromosome 6q16.3, a susceptibility locus for childhood obesity (Meyre et al. 2004). A recent genetic analysis looking for associations of MCHR2 single nucleotide polymorphisms (SNPs) with severe obesity of children implied a possible involvement of MCHR2 in food intake abnormalities in obese children (Ghoussaini et al. 2007). Cynomolgus monkey expresses both MCHR1 and MCHR2 and some become obese with age and a sedentary lifestyle, with parallels to human obesity. Our preliminary data from the cynomolgus monkey study showed an association of antagonism of MCHR1 with a downtrend in several obesity-related parameters. However, the effects of AMG 076 at the doses used were not robust enough to reach statistical significance, particularly so with the effects on IAF and food intake. Further studies with more animals and additional data such as on energy expenditure are needed to be conclusive. If confirmed, our current data with AMG 076 would suggest that antagonism of MCHR1 alone may exert a likely moderate anti-obesity effect in monkeys and potentially in humans and combination with therapies targeting other pathways may be needed. FDA has recently approved Qsynia for treating obesity, which is a combination of two known drugs, phentermine and topiramate. Phentermine is a psychostimulant and topiramate is an anticonvulsant (Cosentino et al. 2011; Holes-Lewis et al. 2013). This may be signaling the start of a new trend of multi-agent combination therapy for obesity. Interestingly, it has been reported that combination of rimonabant (a CB1 receptor antagonist) and SNAP-94,847 (a MCH1 receptor antagonist) was highly effective in reducing body weight in DIO mice below that achieved by either monotherapies (Verty et al. 2013).

A number of MCHR1 antagonists have been developed as potential anti-obesity drugs (Borowsky et al. 2002; Luthin 2007; Gehlert et al. 2009; Jeon and Cheon 2009). Accumulating data strongly support an involvement of the MCH system in regulating behaviors in addition to feeding and body weight homeostasis (Pissios et al. 2006; Antal-Zimanyi and Khawaja 2009; Chung et al. 2011). For example, central administration of MCH increased anxiety in mice (Monzón and De Barioglio 1999; Smith et al. 2006), while MCHR1 knockout mice were less anxious than their wild-type counterparts (Kennedy et al. 2003; Zhou et al. 2005; Roy et al. 2006; Smith et al. 2006). Furthermore, MCHR1 antagonists exhibit anxiolytic and antidepressant activities in rodents (Chaki et al. 2005; David et al. 2007; Gehlert et al. 2009). We were able to recapitulate these findings in our own behavioral analyses of rodents treated with AMG 076 (S. L. Wang, K. Dai, unpubl. data, manuscript in preparation). Whether these effects would also be seen in higher species remains to be determined.

## Abbreviations

AMG 076: 1-[2-((4aR, 11R, 11aS)-11-Methyl-9-trifluoromethyl-1,3,4,4a,5,6,11,11a-octahydro-pyrido[4,3-b]carbazol-2-yl)-ethyl]-cyclohexane carboxylic acid
ANOVA: analysis of variance
BMI: body mass index
CT: computed tomography
DEXA: dual energy x-ray absorptiometry
DIO: diet-induced obese
GPCR: G protein-coupled receptor
IAF: Intra-abdominal fat
MCH: melanin-concentrating hormone
MCH: Melanin-concentrating hormone
MCHR: melanin-concentrating hormone receptor
NHP: nonhuman primates

## Supporting Information

**Table S1.** Dose-Related Plasma Levels of AMG 076 in C57BL/6 Female Mice Fed a High-fat Diet **Table S2.** Dose-Related Plasma Levels of AMG 076 in Obese Cynomolgus Monkeys

## References

[b1] Allison DB, Zannolli R, Narayan KM (1999). The direct health care costs of obesity in the United States. Am J Public Health.

[b3] An S, Cutler G, Zhao JJ, Huang S, Tian H, Li W (2001). Identification and characterization of a melanin concentrating hormone receptor. Proc Natl Acad Sci U S A.

[b4] Antal-Zimanyi I, Khawaja X (2009). The role of melanin-concentrating hormone in energy homeostasis and mood disorders. 2009. J Mol Neurosci.

[b5] Baskin ML, Ard J, Franklin F, Allison DB (2005). Prevalence of obesity in the United States. Obes Rev.

[b6] Bello NT, Liang NC (2011). The use of serotonergic drugs to treat obesity–is there any hope?. Drug Des Devel Ther.

[b7] Bjursell M, Gerdin AK, Ploj K, Svensson D, Svensson L, Oscarsson J (2006). Melanin-concentrating hormone receptor 1 deficiency increases insulin sensitivity in obese leptin-deficient mice without affecting body weight. Diabetes.

[b8] Borowsky B, Durkin MM, Ogozalek K, Marzabadi MR, DeLeon J, Lagu B (2002). Antidepressant, anxiolytic and anorectic effects of a melanin-concentrating hormone-1 receptor antagonist. Nat Med.

[b9] Bronner LL, Kanter DS, Manson JE (1995). Primary prevention of stroke. N Engl J Med.

[b10] Chaki S, Funakoshi T, Hirota-Okuno S, Nishiguchi M, Shimazaki T, Iijima M (2005). Anxiolytic- and antidepressant-like profile of ATC0065 and ATC0175: nonpeptidic and orally active melanin-concentrating hormone receptor 1 antagonists. J Pharmacol Exp Ther.

[b11] Chen Y, Hu C, Hsu CK, Zhang Q, Bi C, Asnicar M (2002). Targeted disruption of the melanin-concentrating hormone receptor-1 results in hyperphagia and resistance to diet-induced obesity. Endocrinology.

[b12] Chen L, Magliano DJ, Zimmet PZ (2011). The worldwide epidemiology of type 2 diabetes mellitus-present and future perspectives. Nat Rev Endocrinol.

[b13] Chung S, Parks GS, Lee C, Civelli O (2011). Recent updates on the melanin-concentrating hormone (MCH) and its receptor system: lessons from MCH1R antagonists. J Mol Neurosci.

[b14] Cosentino G, Conrad AO, Uwaifo GI (2011). Phentermine and topiramate for the management of obesity: a review. Drug Des Devel Ther.

[b15] David DJ, Klemenhagen KC, Holick KA, Saxe MD, Mendez I, Santarelli L (2007). Efficacy of the MCHR1 antagonist N-[3-(1-{[4-(3,4-difluorophenoxy)phenyl]methyl}(4-piperidyl))-4-methylphenyl]-2-methylpropanamide (SNAP 94847) in mouse models of anxiety and depression following acute and chronic administration is independent of hippocampal neurogenesis. J Pharmacol Exp Ther.

[b17] Della-Zuana O, Presse F, Ortola C, Duhault J, Nahon JL, Levens N (2002). Acute and chronic administration of melanin-concentrating hormone enhances food intake and body weight in Wistar and Sprague-Dawley rats. Int J Obes Relat Metab Disord.

[b18] Gehlert DR, Rasmussen K, Shaw J, Li X, Ardayfio P, Craft L (2009). Preclinical evaluation of melanin-concentrating hormone receptor 1 antagonism for the treatment of obesity and depression. J Pharmacol Exp Ther.

[b19] Ghoussaini M, Vatin V, Lecoeur C, Abkevich V, Younus A, Samson C (2007). Genetic study of the melanin-concentrating hormone receptor 2 in childhood and adulthood severe obesity. J Clin Endocrinol Metab.

[b20] Gomori A, Ishihara A, Ito M, Mashiko S, Matsushita H, Yumoto M (2003). Chronic intracerebroventricular infusion of MCH causes obesity in mice: melanin-concentrating hormone. Am J Physiol Endocrinol Metab.

[b21] Hervieu G (2003). Melanin-concentrating hormone functions in the nervous system: food intake and stress. Expert Opin Ther Targets.

[b22] Hill J, Duckworth M, Murdock P, Rennie G, Sabido-David C, Ames RS (2001). Molecular cloning and functional characterization of MCHR2, a novel human MCH receptor. J Biol Chem.

[b23] Holes-Lewis KA, Malcolm R, O'Neil PM (2013). Pharmacotherapy of obesity: clinical treatments and considerations. Am J Med Sci.

[b24] Institute for Laboratory Animal Resources (1996). Guide for the Care and Use of Laboratory Animals.

[b25] Ito M, Ishihara A, Gomori A, Matsushita H, Ito M, Metzger JM (2010). Mechanism of the anti-obesity effects induced by a novel melanin-concentrating hormone 1-receptor antagonist in mice. Br J Pharmacol.

[b26] Jeon MK, Cheon HG (2009). Promising strategies for obesity pharmacotherapy: melanocortin-4 (MC-4) receptor agonists and melanin concentrating hormone (MCH) receptor-1 antagonists. Curr Top Med Chem.

[b27] Jeon JY, Bradley RL, Kokkotou EG, Marino FE, Wang X, Pissios P (2006). MCH-/-mice are resistant to aging-associated increases in body weight and insulin resistance. Diabetes.

[b28] Kennedy AR, Todd JF, Dhillo WS, Seal LJ, Ghatei MA, O'Toole CP (2003). Effect of direct injection of melanin-concentrating hormone into the paraventricular nucleus: further evidence for a stimulatory role in the adrenal axis via SLC-1. J Neuroendocrinol.

[b29] Kokkotou E, Jeon JY, Wang X, Marino FE, Carlson M, Trombly DJ (2005). Mice with MCH ablation resist diet-induced obesity through strain-specific mechanisms. Am J Physiol Regul Integr Comp Physiol.

[b30] Kolakowski LF, Jung BP, Nguyen T, Johnson MP, Lynch KR, Cheng R (1996). Characterization of a human gene related to genes encoding somatostatin receptors. FEBS Lett.

[b31] Ludwig DS, Tritos NA, Mastaitis JW, Kulkarni R, Kokkotou E, Elmquist J (2001). Melanin-concentrating hormone overexpression in transgenic mice leads to obesity and insulin resistance. J Clin Invest.

[b32] Luthin DR (2007). Anti-obesity effects of small molecule melanin-concentrating hormone receptor 1 (MCHR1) antagonists. Life Sci.

[b33] Marsh DJ, Weingarth DT, Novi DE, Chen HY, Trumbauer ME, Chen AS (2002). Melanin-concentrating hormone 1 receptor-deficient mice are lean, hyperactive, and hyperphagic and have altered metabolism. Proc Natl Acad Sci U S A.

[b34] Meyre D, Lecoeur C, Delplanque J, Francke S, Vatin V, Durand E (2004). A genome-wide scan for childhood obesity-associated traits in French families shows significant linkage on chromosome 6q22.31-q23.2. Diabetes.

[b35] Mihalic JT, Fan P, Chen X, Chen X, Fu Y, Motani A (2012). Discovery of a novel melanin concentrating hormone receptor 1 (MCHR1) antagonist with reduced hERG inhibition. Bioorg Med Chem Lett.

[b36] Mizuno TM, Kleopoulos SP, Bergen HT, Roberts JL, Priest CA, Mobbs CV (1998). Hypothalamic pro-opiomelanocortin mRNA is reduced by fasting and [corrected] in ob/ob and db/db mice, but is stimulated by leptin. Diabetes.

[b37] Monzón ME, De Barioglio SR (1999). Response to novelty after i.c.v. injection of melanin-concentrating hormone (MCH) in rats. Physiol Behav.

[b38] Nahon JL (2006). The melanocortins and melanin-concentrating hormone in the central regulation of feeding behavior and energy homeostasis. C R Biol.

[b39] Pissios P, Bradley RL, Maratos-Flier E (2006). Expanding the scales: the multiple roles of MCH in regulating energy balance and other biological functions. Endocr Rev.

[b40] Powell AG, Apovian CM, Aronne LJ (2011). New drug targets for the treatment of obesity. Clin Pharmacol Ther.

[b41] Presse F, Sorokovsky I, Max JP, Nicolaidis S, Nahon JL (1996). Melanin-concentrating hormone is a potent anorectic peptide regulated by food-deprivation and glucopenia in the rat. Neuroscience.

[b42] Qu D, Ludwig DS, Gammeltoft S, Piper M, Pelleymounter MA, Cullen MJ (1996). A role for melanin-concentrating hormone in the central regulation of feeding behaviour. Nature.

[b43] Rossmeisl M, Rim JS, Koza RA, Kozak LP (2003). Variation in type 2 diabetes–related traits in mouse strains susceptible to diet-induced obesity. Diabetes.

[b44] Roy M, David NK, Danao JV, Baribault H, Tian H, Giorgetti M (2006). Genetic inactivation of melanin-concentrating hormone receptor subtype 1 (MCHR1) in mice exerts anxiolytic-like behavioral effects. Neuropsychopharmacology.

[b45] Sailer AW, Sano H, Zeng Z, McDonald TP, Pan J, Pong SS (2001). Identification and characterization of a second melanin-concentrating hormone receptor, MCH-2R. Proc Natl Acad Sci U S A.

[b46] Saito Y, Nothacker HP, Civelli O (2000). Melanin-concentrating hormone receptor: an orphan receptor fits the key. Trends Endocrinol Metab.

[b47] Shimada M, Tritos NA, Lowell BB, Flier JS, Maratos-Flier E (1998). Mice lacking melanin-concentrating hormone are hypophagic and lean. Nature.

[b48] Smith DG, Davis RJ, Rorick-Kehn L, Morin M, Witkin JM, McKinzie DL (2006). Melanin-concentrating hormone-1 receptor modulates neuroendocrine, behavioral, and corticolimbic neurochemical stress responses in mice. Neuropsychopharmacology.

[b49] Stricker-Krongrad A, Dimitrov T, Beck B (2001). Central and peripheral dysregulation of melanin-concentrating hormone in obese Zucker rats. Brain Res Mol Brain Res.

[b50] Tan CP, Sano H, Iwaasa H, Pan J, Sailer AW, Hreniuk DL (2002). Melanin-concentrating hormone receptor subtypes 1 and 2: species-specific gene expression. Genomics.

[b100] Tritos NA, Mastaitis JW, Kokkotou E, Maratos-Flier E (2001). Characterization of melanin concentrating hormone and preproorexin expression in the murine hypothalamus. Brain Res.

[b51] Verty ANA, Lockie SH, Stefanidis A, Oldfield BJ (2013). Anti-obesity effects of the combined administration of CB1 receptor antagonist rimonabant and melanin-concentrating hormone antagonist SNAP-94847 in diet-induced obese mice. Int J Obes.

[b52] Vetter ML, Faulconbridge LF, Webb VL, Wadden TA (2010). Behavioral and pharmacologic therapies for obesity. Nat Rev Endocrinol.

[b53] Wright SM, Aronne LJ (2011). Obesity in 2010: the future of obesity medicine: where do we go from here?. Nat Rev Endocrinol.

[b54] Zheng H, Patterson LM, Morrison C, Banfield BW, Randall JA, Browning KN (2005). Melanin concentrating hormone innervation of caudal brainstem areas involved in gastrointestinal functions and energy balance. Neuroscience.

[b55] Zhou D, Shen Z, Strack AM, Marsh DJ, Shearman LP (2005). Enhanced running wheel activity of both Mch1r- and Pmch-deficient mice. Regul Pept.

