# Supplementary material for: Evaluation of AMG 076, a potent and selective MCHR1 antagonist, in rodent and primate obesity models
Source: Pharmacol Res Perspect. 2013 Sep 17;1(1):e00003. doi: 10.1002/prp2.3 (PMC4184568; doi:10.1002/prp2.3)
Supplement: Supplementary file 1 — Table S1. Dose-Related Plasma Levels of AMG 076 in C57BL/6 Female Mice Fed a High-fat Diet Table S2. Dose-Related Plasma Levels of AMG 076 in Obese Cynomolgus Monkeys [file prp20001-e00003-sd1.docx]

**Appendix**

**Supplemental Data**

Evaluation of AMG 076, a Potent and Selective MCHR1 Antagonist, in Rodent and Primate Obesity Models

Alykhan S. Motani (Ph.D.), Jian Luo (Ph.D.), Lingming Liang (M.S.), Jeff T. Mihalic (Ph.D.), Xiaoqi Chen (Ph.D.), Leping Li (Ph.D.), Liang Tang (Ph.D.), Juan Jaen (Ph.D.), Jin-Long Chen (Ph.D.), Kang Dai (Ph.D.)

**Table 1. Dose Related Plasma Levels of AMG 076 in C57BL/6 Female Mice Fed a High-fat Diet**

|  | 0.1 mg/kg | 0.3 mg/kg | 1 mg/kg | 3 mg/kg | 10 mg/kg |
| --- | --- | --- | --- | --- | --- |
| AUC (μg h/L) | 184 | 638 | 1960 | 4800 | 15500 |
| Cmax (μg /L) | 14 | 51 | 186 | 472 | 1980 |

AUC (0-24 hr) = area under the concentration curve, C_max_ = maximum observed concentration, data are represented as mean of n = 10 mice per group.

Table 2. Dose Related Plasma Levels of AMG 076 in Obese Cynomolgus Monkeys

|  | 1 mg/kg | | 3 mg/kg | | 10 mg/kg | |
| --- | --- | --- | --- | --- | --- | --- |
| Time after dosing | 2 hr | 3 hr | 2 hr | 3 hr | 2 hr | 3 hr |
| Mean (μg/L) | 465 | 421 | 751 | 854 | 909 | 872 |

Data are represented as mean of n = 6 monkeys per group.
